# Rapid Determination of Geniposide and Baicalin in Lanqin Oral Solution by Near-Infrared Spectroscopy with Chemometric Algorithms during Alcohol Precipitation

**DOI:** 10.3390/molecules28010004

**Published:** 2022-12-20

**Authors:** Hui Ma, Ming Chen, Siyu Zhang, Hongye Pan, Yong Chen, Yongjiang Wu

**Affiliations:** 1 College of Pharmaceutical Sciences, Zhejiang University, Hangzhou 310058, China; 2 Shandong Academy of Pharmaceutical Sciences, Jinan 250098, China; 3 Department of Clinical Pharmacy, The First Affiliated Hospital, Zhejiang University School of Medicine, Hangzhou 310003, China

**Keywords:** lanqin oral solution, process analysis, chemometrics, variable selection, spectroscopy, variable combination population analysis-iteratively retaining informative variables

## Abstract

The selection of key variables is an important step that improves the prediction performance of a near-infrared (NIR) real-time monitoring system. Combined with chemometrics, NIR spectroscopy was employed to construct high predictive accuracy, interpretable models for the rapid detection of the alcohol precipitation process of Lanqin oral solution (LOS). The variable combination population analysis-iteratively retaining informative variables (VCPA-IRIV) was innovatively introduced into the variable screening process of the model of geniposide and baicalin. Compared with the commonly used synergy interval partial least squares regression, competitive adaptive reweighted sampling, and random frog, VCPA-IRIV achieved the maximum compression of variable space. VCPA-IRIV-partial least squares regression (PLSR) only needs to use about 1% of the number of variables of the original data set to construct models with Rp values greater than 0.95 and RMSEP values less than 10%. With the advantages of simplicity and strong interpretability, the prediction ability of the PLSR models had been significantly improved simultaneously. The VCPA-IRIV-PLSR models met the requirements of rapid quality detection. The real-time detection system can help researchers to understand the quality rules of geniposide and baicalin in the alcohol precipitation process of LOS and provide a reference for the optimization of a LOS quality control system.

## 1. Introduction

Lanqin oral solution (LOS) is a clinical traditional Chinese medicine (TCM) for the treatment of pharyngitis, mainly made of *Isatidis Radix*, *Gardeniae Fructus*, *Scutellariae Radix*, *Phellodendri Chinensis Cortex,* and *Sterculiae Lychnophorae Semen*. Due to their specific pharmacological activities, geniposide and baicalin are treated as quality control indicators [1,2]. Alcohol precipitation has been regarded as a typical method for the purification of TCM [3]. Alcohol precipitation is the only purification process during the production of LOS. The current process control method of alcohol precipitation relies heavily on onsite human experience. A lack of knowledge of the sample quality during the production process ultimately leads to fluctuations in the quality of the final product.

In order to enhance the efficiency of processes, and ensure the quality and stability of products, the process analytical technology (PAT) industrial guideline was first published by the Food and Drug Administration (FDA) in 2004 [4]. The aim of PAT was to achieve process variables real-time monitoring and provide instruction for process conditions modification. PAT is gradually introduced into the TCM manufacturing industry [5,6,7], however, traditional chemical analysis methods such as high-performance liquid chromatography (HPLC), and ultraviolet and visible spectrophotometry are time-consuming [8].

Near-infrared (NIR) spectroscopy, with its features of being fast, nondestructive, and efficient, has been an ideal PAT tool in multiple fields since the end of the 20th century [9,10,11,12]. In most research, samples can be analyzed by NIRs without pretreatment [13,14,15]. In the meanwhile, NIR spectra can provide effective physical and chemical information on multiple active ingredients, in accordance with the requirements of multi-indexes in the complex TCM [16]. However, due to the high-dimensional data and the existence of interference information, it is difficult to analyze the spectra directly.

In order to correlate critical quality attributes (CQAs) with spectral information, NIRs need to be analyzed in combination with chemometrics in general [17,18]. In chemometric processing, variable selection is an important step to reduce modeling difficulty while improving model prediction accuracy [19]. By evaluating and selecting wave points, irrelevant information will be efficiently identified and eliminated from the spectral matrix, thereby extracting effective information from high dimensional data [20]. The interpretability of the model will be enhanced while reducing the interference information. At present, a number of variable selection methods have been proposed, however, none of them are suitable for all data sets. It is necessary to select the most suitable variable selection method for the data set by comparing the model performance parameters.

In this study, four kinds of variable selection methods were innovatively introduced into the LOS quality monitoring system. The pharmacologically active ingredients of the LOS, namely geniposide and baicalin, were considered as CQAs. Various preprocessing methods were compared to remove noise in the spectra. Synergy interval partial least squares regression (SIPLS), competitive adaptive reweighted sampling (CARS), random frog (RF), and variable combination population analysis-iteratively retaining informative variables (VCPA-IRIV) were systematically compared to screen the key spectral variables associated with CQAs. The constructed optimal local models can be applied to realize the process analysis of alcohol precipitation of LOS.

## 2. Results and Discussion

### 2.1. Reference Data Analysis

**Figure 1:**
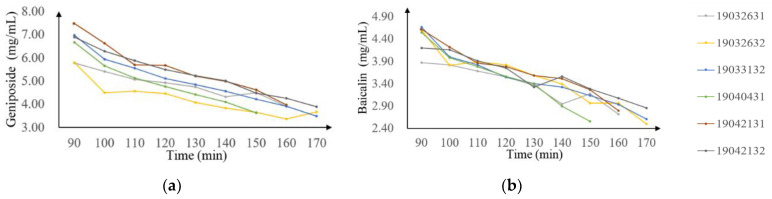
Concentration dynamics of 2 chosen marking components during alcohol precipitation. (**a**) geniposide; (**b**) baicalin.

Geniposide and baicalin were fully separated by HPLC. The dynamic curves for two marking components during the alcohol precipitation are displayed in Figure 1. The concentration of geniposide and baicalin was distributed uniformly in the range of 2.74–7.82 mg/mL and 1.76–4.72 mg/mL, respectively. The time evolution curves of the concentration of the two CQAs had similar trends, obviously. With the addition of the alcohol solution, the concentration of substances in the system decreased continuously. But at the same time, it can also be found that the time required for each batch to reach the end of the process was inconsistent. The endpoint of the alcohol precipitation process depends on the alcohol content of the solvent system. For example, the alcohol content of the production batch 19040431 of LOS reached the standard at the 150th minute, while the batch 19042131 sample took 170 min. The process gap was caused by the batch-to-batch fluctuation of the input materials and other process parameters depending on the worker’s experience, such as the rate of addition of the alcohol solution. These factors also caused obvious fluctuations in the concentration of CQAs in different batches of LOS, which demonstrated the importance of process analysis.

### 2.2. Raw Spectral Data Analysis

**Figure 2:**
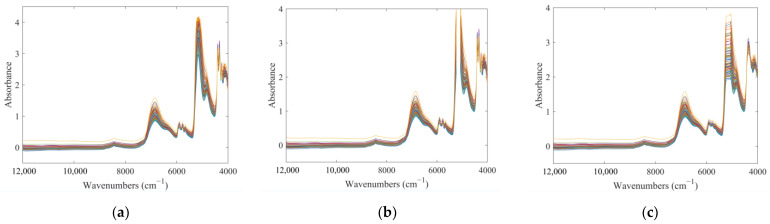
NIR absorbance spectra. (**a**) the raw NIR absorbance spectra; (**b**) processed spectra; (**c**) SG smoothing processed spectra. Different colors represent different samples.

The raw NIR absorbance spectra are shown in Figure 2a. The combined physical and chemical information of the sample was collected in the NIR absorbance spectra. The wide absorption band in the range of 7680–7540 cm^−1^ was mainly generated by the hydrogen-bonded O-H and the most obvious absorption peak near 5160 cm^−1^ was attributed to the O-H group of solvent. These two bands were typical peaks of the TCM aqueous alcoholic solution. The first combined absorption of the C-H group occurred in the region of 4413–4000 cm^−1^, which belongs to the third spectral region. At the same time, it can be found that the spectra in this region 5235–5078 cm^−1^ showed obvious supersaturated absorption, so the variables in this region were removed to avoid affecting the subsequent analysis. The spectra, after removing the supersaturated region, are shown in Figure 2b. The spectra of different samples were similar and difficult to analyze directly. Therefore, it was necessary to introduce chemometric methods for the information mining of spectra.

### 2.3. Division of Samples and Spectral Pre-Treatment

**Figure 3:**
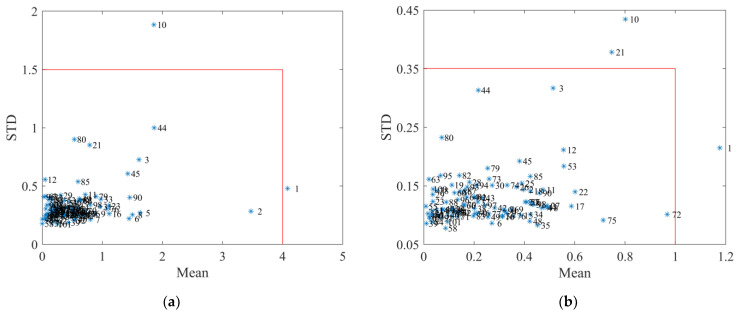
The results of MCCV (**a**) geniposide; (**b**) baicalin. Blue asterisks represent different samples.

The results of Monte Carlo cross-validation (MCCV) outlier elimination are shown in Figure 3. For geniposide, the sample points with mean prediction residuals greater than 4, and STD value greater than 1.5, were identified as outlier samples, namely samples No. 1 and No. 10. For baicalin, the mean prediction errors of samples No. 1, 10, and 21 were greater than one, or the STD value was greater than 0.35. Combining the screening results of the two indicators, samples No. 1 and No. 10 were identified as abnormal samples and were removed before subsequent modeling steps.

The datasets were divided into calibration sets and prediction sets by sample set partitioning based on joint x-y distance (SPXY) at a ratio of 3∶1. The concentration ranges of samples for the calibration set and prediction set are listed in Table 1. As shown in Table 1, the content ranges of target components in calibration sets covered the range in the prediction sets, which was beneficial for the stability and robustness of established models.

The modeling results of two indexes by different pretreatment methods are listed in Table 2. Compared with normalization, standard normal variate transformation (SNV) and multiplicative scatter correction (MSC), Savitzky-Golay (SG) smoothing preprocessed spectra were optimal for model construction. The spectra after SG smoothing are shown in Figure 2c. It can be found that, compared with the original spectra, the spectral region with the high noise, such as the spectral region from 4413 to 4000 cm^−1^, became significantly smoother. The correlation coefficients of cross-validation (Rcv) values of the pretreated models of geniposide and baicalin improved from 0.9032 and 0.8847 to 0.9151 and 0.9187, respectively. At the same time, the prediction error of the model decreased, and the residual predictive deviation of cross-validation (RPDCV) values increased. The improvements of these model performance parameters also proved that the SG smoothing preprocessing removed the noise in the raw spectra, reduced the interference in the modeling process, and achieved a more accurate prediction ability for indicators.

### 2.4. SIPLS Wavelength Interval Selection Process

During the selection of SIPLS, the 2032 variables in the full spectrum were sequentially divided into 10 to 30 equal-length subintervals and the subintervals were combined. The optimal subinterval combination method, and setting of LVs for partial least squares regression (PLSR), were chosen according to the lowest RMSECV. The performance parameters of the optimal local PLSR models obtained under different segmentation conditions are listed in Table 3. It can be observed that for the geniposide model, the interval division of 10–30 can optimize the model, and the RMSECV values of the obtained local models were all lower than 0.4782 of the global model. For baicalin, the optimization of interval segmentation was more necessary. Compared with the RMSECV value of 0.2567 of the original model, only when the number of segmentations was 27 or 28 did the error value of the model prediction decrease. Finally, the optimal interval separation numbers for geniposide and baicalin were 26 and 28, and the number of modeling variables was compressed from 2032 to 312 and 289, respectively. The difference in the changes in model performance after SIPLS treatment proved that the method of wavelength interval selection was suitable for the geniposide dataset but less applicable to the baicalin dataset. It was necessary to choose an appropriate variable selection method according to the characteristics of each dataset.

### 2.5. CARS Wavenumber Selection Process

**Figure 4:**
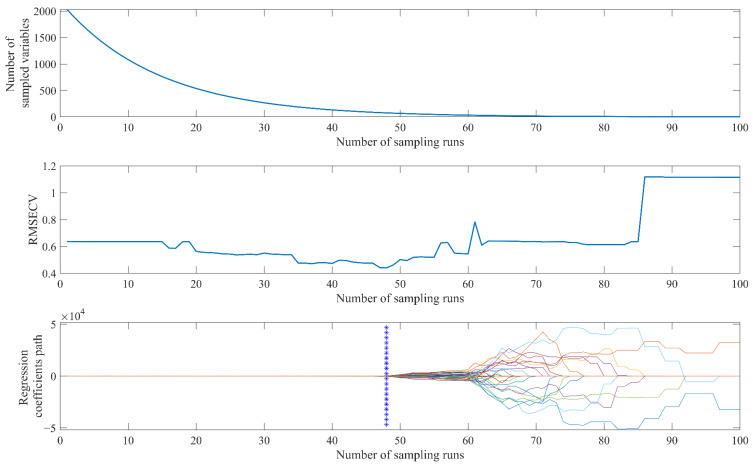
CARS variable selection on geniposide dataset.

The screening process of the key variables of geniposide-based CARS is shown in Figure 4. A total of 100 Monte Carlo samplings were carried out. In the process of the first 30 sampling modeling, the number of variables dropped sharply, the variables were effectively removed, and then the rate of decline of the variables decreased. The two selection processes of fast selection and refined selection of variables can quickly eliminate wave points with small absolute regression coefficients. The RMSECV values of the local models constructed during the operation showed a decreasing trend in the early sampling stage, which proved that a number of irrelevant variables were eliminated. After the 48th run, irrelevant variables had been removed and CARS began to eliminate effective variables related to the target index. Therefore, the RMSECV value increased rapidly. The relevant model performance parameters of the PLSR model constructed by the best subset of variables identified by CARS are listed in Table 4. The number of variables of geniposide and baicalin datasets was reduced from 2032 to 76 and 38, respectively. The number of variables was effectively compressed by CARS. At the same time, compared with the global models, the predictive ability of the models constructed by the retained key variables was significantly improved.

### 2.6. RF Wavenumber Selection Process

**Figure 5:**
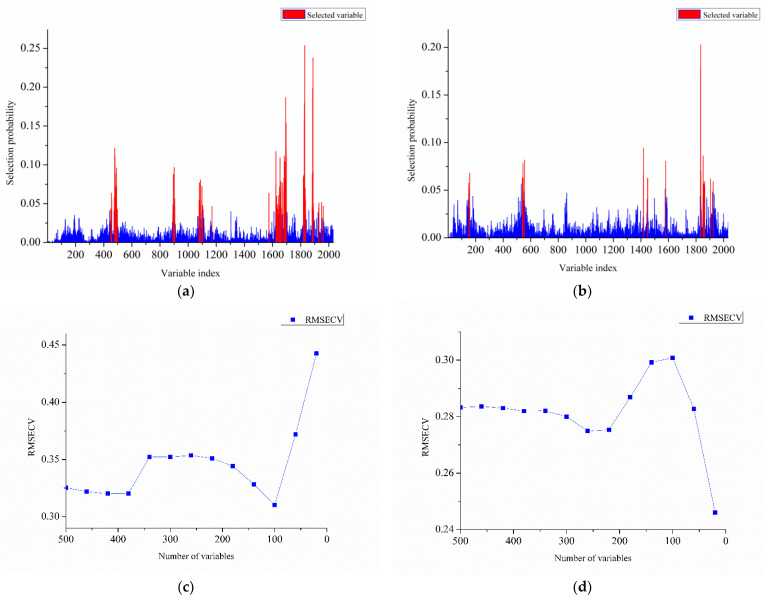
RF variable selection on dataset: (**a**) selection probability for each variable calculated for geniposide dataset; (**b**) selection probability for each variable calculated for baicalin dataset; (**c**) relationship between RMSECV value and number of variables of geniposide dataset (**d**) relationship between RMSECV value and number of variables of baicalin dataset.

The number of variables in the geniposide and baicalin datasets was compressed to 100 and 20 after RF screening. The distribution of the key variables identified by RF over the variable space is shown in Figure 5a,b. For two indicators, the probability of 2032 variables being selected by the 10,000 local models fluctuated in the range of 0–25%. It can be found that compared with baicalin, the geniposide dataset had more wave points with high probability. The further optimization process of selecting 500 high-probability wave points is shown in Figure 5c,d. For geniposide, the elimination of 400 lower probability variables improved the predictive ability of the model. When the number of variables was further reduced to 60, the predictive ability of the model decreased rapidly. It was proved that for geniposide, the top 100 highly selective wave points contributed to the construction of the model, which was also consistent with the fact that its variables generally had a higher selection probability. The RPDCV value of the geniposide model was improved from 2.48 to 3.79 after RF optimization. For baicalin, the removal of the first 400 wave points had little effect on the model performance. The RMSECV value of the baicalin prediction model decreased rapidly during the removal of the last 100 variables. It represented that the baicalin model was mainly affected by wave points with the highest selection probability. By retaining the 20 variables with the highest selection probability, the predictive ability of the baicalin model was significantly improved, and the RPDCV value increased from 2.53 to 3.06.

### 2.7. VCPA-IRIV Wavenumber Selection Process

**Figure 6:**
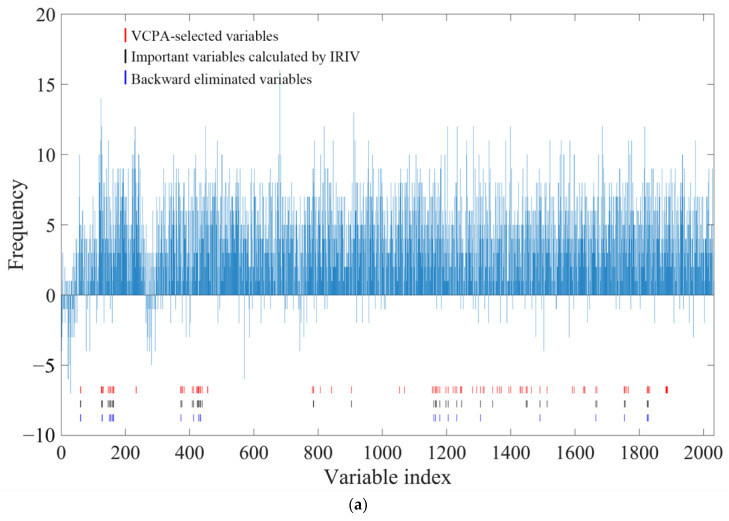

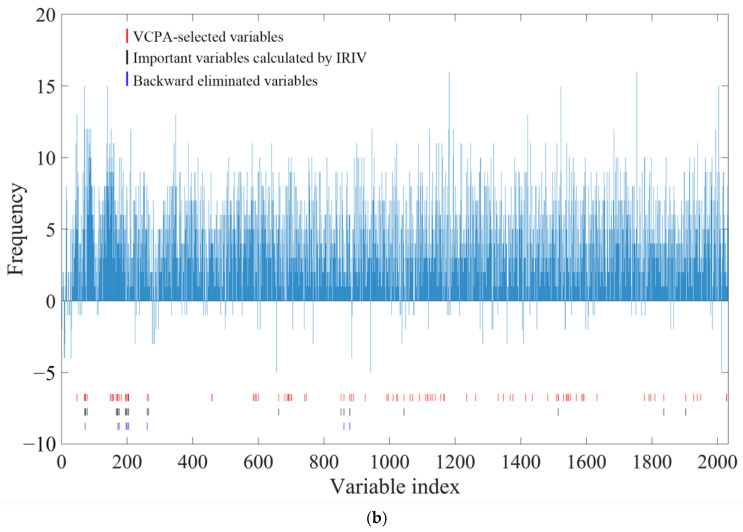
VCPA-IRIV variable selection on dataset (**a**) geniposide; (**b**) baicalin.

The process of VCPA-IRIV evaluation is shown in Figure 6. The frequency histogram in the figure represents the frequency of 2032 variables selected by the top 15% of the best models or the 5% of the worst models when VCPA was first run. A positive frequency meant that the wave point was more likely to be selected by the best model, while a negative frequency represented that the variable was more likely to be included by the worst model. It can be found that each wave point showed a certain trend. The variables corresponding to the red lines in the figure were the 100 high-contribution variables screened by VCPA. Running IRIV with these 100 variables as input achieved further optimization and simplification of the model. In the key variable selection of geniposide, a total of 48 wave points were identified as strongly informative or weakly informative variables (the variables corresponding to the black lines in the figure). In the subsequent backward elimination, after the wave points corresponding to the 27 blue lines in the figure were removed, the prediction error of the local model was further reduced. Therefore, there were a total of 21 variables retained for subsequent modeling for the geniposide spectral dataset after calculation by VCPA-IRIV. For baicalin, VCPA also extracted 100 wave points for the calculation of IRIV. Twenty-three variables were identified as uninformative and interfering variables by IRIV. Ten variables were confirmed to be eliminated in the backward elimination strategy, which was more beneficial to the performance of the model. A total of 13 wave points were retained in the baicalin data set for the subsequent construction of the PLSR model. The Rcv values of the geniposide and baicalin local models constructed by the variables selected by VCPA-IRIV increased from 0.9151 and 0.9187 to 0.9883 and 0.9502, respectively. The RPDCV values of the models were improved to more than three, while the RMSECV values were reduced to 0.1793 and 0.2329, respectively. The improvements in the model parameters proved that the variables selected by VCPA-IRIV were beneficial to the construction of quantitative models.

### 2.8. Comparison of Different Variable Selection Methods

**Figure 7:**
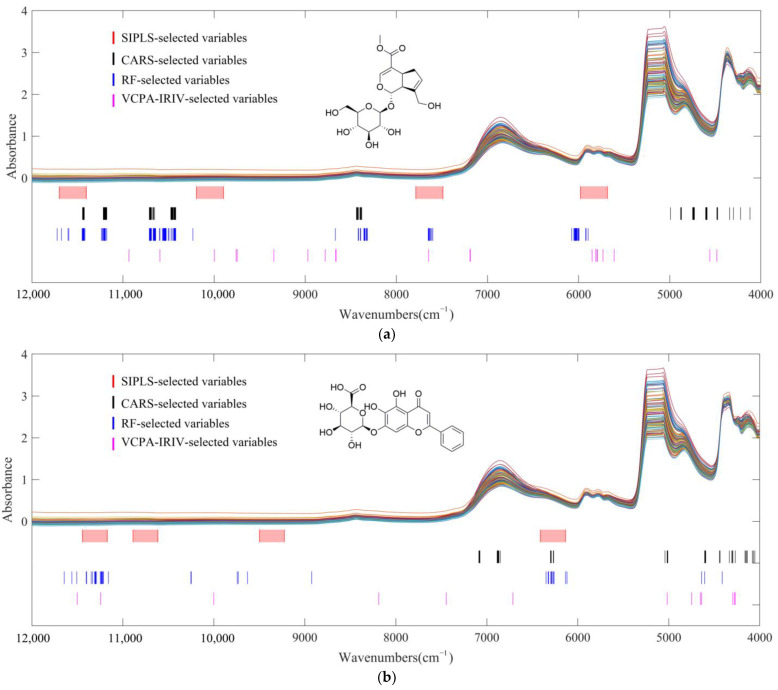
The optimal subset of variables selected by different variable selection methods. (**a**) geniposide; (**b**) baicalin.

The key variables determined by the 4 variable screening methods are shown in Figure 7. It can be observed that although the principles of the four methods were different, the distribution ranges of the final selected key variables were similar. A major feature of the structure of geniposide was that it contained multiple hydroxyl groups. Therefore, the absorption regions above 10,000 cm^−^^1^ and 5000–4000 cm^−^^1^ were selected in relation to the vibration of -OH. The absorption near 7600 cm^−^^1^ and 6000 cm^−^^1^ corresponded to the basic structure of organic compounds, second and first overtone absorption of the C-H bond. For baicalin, the high selectivity of the variables located in the region of 5000–4000 cm^−^^1^ and above 10,000 cm^−^^1^ was also derived from its polyhydroxy substance structure. Different from geniposide, the absorption performance of C-H of baicalin moved to around 6500 cm^−^^1^, which came from the influence of the benzene ring structure. The first overtone absorption of the C-H of C with sp2 hybridization was higher than one of sp3 hybridization [21]. The key variables selected by the four methods correspond to the structures of CQAs.

It was observed from the parameters in Table 2 that compared with the other three methods, the VCPA-IRIV selection achieved the greatest compression of the variable space while obtaining the optimal quantitative model. The process of backward elimination guaranteed the predictive ability of the model established by VCPA-IRIV selection. It can also be observed in Figure 7 that the 21 and 13 key variables screened by VCPA-IRIV were distributed in the key regions discussed above. It proved that VCPA-IRIV not only achieved efficient compression of variable space but also took into account the retention of effective spectral information.

**Figure 8:**
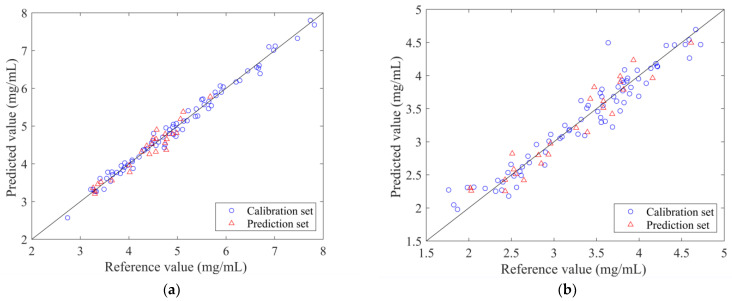
Scatter plot of reference measurements and NIR prediction. (**a**) geniposide; (**b**) baicalin.

The 25 samples of the divided prediction set were introduced to evaluate the accuracy of the VCPA-IRIV-PLSR models. The observed and predicted values of the VCPA-IRIV-PLSR models for the 99 LOS samples are shown in Figure 8. It can be observed that the predicted values of the optimal models were well correlated with the measured values. The sample points were evenly distributed around y = x. The model parameters corresponding to the calibration set and prediction set samples are shown in Table 5. The root-mean-square error of prediction (RMSEP) values of the established optimal models were low and close to the root-mean-square error of calibration (RMSEC) values. The Rp values of the geniposide and baicalin models reached 0.9654 and 0.9597, respectively. The obtained models had RPD values greater than three. These model parameters proved that the prediction accuracy of the PLSR models was high and met the requirements of the application. The quantitative prediction model optimized by VCPA-IRIV had the advantages of simple structure, and at the same time improved the prediction accuracy of the model. The VCPA-IRIV-PLSR models can be applied to the rapid quality detection of geniposide and baicalin during the alcohol precipitation process of LOS.

## 3. Materials and Methods

### 3.1. Materials and Reagents

Alcohol precipitation samples were collected from one sampling point of the enterprise production site of Yangtze River Pharmaceutical Group (Taizhou, China). Sampling was started after the 1.5th hour of the alcohol precipitation process and sampled at intervals of 10 min. In a total of 6 batches, 50 samples were obtained. To ensure the representativeness of the sample set and expand the sample size, 51 additional samples were collected randomly during the whole process.

Standard geniposide (≥99%) and baicalin (≥98%) were purchased from Chengdu Must Bio-Technology Co., Ltd. (Chengdu, China). HPLC-grade methanol, acetonitrile, and phosphoric acid were obtained from Merck (Darmstadt, Germany). Analytical grade anhydrous sodium dihydrogen phosphate was used for the mobile phase. Deionised water was purified using a Milli-Q purification system (Millipore, Bedford, MA, USA).

### 3.2. Reference Method

An HPLC method was developed to quantitatively determine the concentration of geniposide and baicalin. Each sample collected from the product line was required to be diluted 10 times with methanol, and then the fluid was filtrated through a 0.45 µm syringe filter. The analysis was performed on Agilent 1200 HPLC system coupled with a diode array detector (DAD). A Luna^®^ C18 column (250 × 4.6 mm, 5 μm) was employed, and the column temperature was maintained at 30 °C. The mobile phase was composed of acetonitrile (mobile phase A) and 0.017 mol/L sodium dihydrogen phosphate solution (add phosphoric acid to adjust the pH to 3, mobile phase B). The gradient elution profile started with A–B (2:98, *v*/*v*) in the first 3 min, and mobile phase A was gradually increased to 20% and 45% at 13 and 35 min. The flow rate was 1.0 mL/min, and the injection volume was 10 μL. The wavelength of the detector was set at 238 nm and 280 nm for geniposide and baicalin, respectively. HPLC chromatograms of standard solution and LOS sample are showed in Figure S1.

### 3.3. NIR Instrument and Data Acquisition

NIR spectra of the LOS alcohol precipitation samples were obtained by a Bruker Matrix-F Fourier transform NIR spectrometer (Bruker Optics Inc., Ettlingen, Germany) with a 2 mm pathlength transmission probe. Spectra were collected from 12,000 to 4000 cm^−1^ and 8 cm^−1^ resolution in absorbance mode at room temperature. Each spectrum was the average of 32 scans and the average spectrum of 4 times measurements was used.

### 3.4. Outlier Elimination

Anomalous samples can lead to a decrease in the predictive accuracy of the NIR model. Therefore, MCCV was applied to detect and eliminate anomalous samples [22]. Calibration sets and prediction sets were randomly partitioned at a ratio of 75:25 to establish PLSR models by Monte Carlo random sampling. The procedure was repeated 1000 times. Then the mean and variance of prediction residuals for each sample were calculated. Finally, according to the discretization of values, abnormal samples with mean and variance that deviated significantly from the group were detected and removed from the data set.

### 3.5. Division of Samples and Spectral Preprocessing

The samples were divided into calibration and prediction sets at the ratio of 3:1 by the SPXY algorithm. Using the x and y variables simultaneously, the distance between samples was calculated to ensure the maximum representation of sample distribution by SPXY [23].

Irrelevant information caused by interfering variables, such as electrical noise and sample background, was contained in the spectra inevitably. When modeling with chemometric methods, it is critical to perform pretreatment that aims at eliminating spectral data-independent information and noise [24]. In this study, normalization, MSC, SNV, SG smoothing, and the combination were employed to eliminate the effects of independent environment variables.

### 3.6. Wavenumber Variables Selection Methods

SIPLS is an improved algorithm based on interval partial least squares regression. When performing SIPLS, the full spectral region is first divided into a certain number of equidistant subintervals, and then these subintervals are combined with all possible permutations. The performance of the local models constructed by the combined spectral intervals was evaluated by the RMSECV value, and the optimal intervals were determined [25]. SIPLS is a commonly applied method for selecting wavelength regions. In this paper, the number of divided intervals was optimized in the range of 10–30, and the number of combinations of intervals was set as 4.

CARS evaluates the importance of variables by the absolute value of the regression coefficients of the local PLSR model. Simulating the survival of the fittest principle of Darwin’s theory of evolution, CARS applies exponentially decreasing function (EDF) and adaptive reweighted sampling (ARS) to forcefully remove wave points with relatively small absolute regression coefficients through multiple modeling and evaluation. At the same time, cross validation was introduced to evaluate the predictive ability of the local model. Finally, the subset of variables with the lowest RMSECV was obtained [26].

RF confirms the regularity of fixed-dimensional and transdimensional moves by building multiple local models. Thus, the probability of each variable being selected is calculated to evaluate the correlation between the wave point and the predictive performance of the model. Research has shown that RF efficiently realizes a search in the model space, and achieves the compression of the variable space. In this experiment, the first 500 variables with high probability were selected as the initial variable subset, and the wave points with relatively low probability were gradually removed with a step value of 40 to establish local models. The variables of the subset corresponding to the model with the lowest RMSECV value were selected as the optimal variables [27].

IRIV implements the importance ranking of variables by building numbers of local models to calculate the impact of each wave point’s inclusion and exclusion on model performance. During the operation of IRIV, the uninformative and interfering variables are first identified and deleted to form a variable subset consisting of strongly informative and weakly informative variables. Then, further backward elimination will be performed on the obtained subset of variables. In this step, it will be confirmed whether a wave point needs to be deleted according to the change of the RMSECV value of the local model created by removing the variable. IRIV has shown robust predictive capabilities in multiple datasets, but its reliance on local models also poses a computationally expensive drawback [28]. Therefore, Xu et al. proposed that VCPA could be introduced to optimize the calculation of IRIV [29]. Combining the advantages of EDF, binary matrix sampling (BMS), and model population analysis (MPA), VCPA can identify irrelevant variables before IRIV calculation, and shrink the variable space.

### 3.7. PLSR

PLSR is a classic linear calibration method that has been widely applied in many types of research. The performance of models is extremely affected by the number of LVs. In this study, leave-one-out cross-validation was used to determine the appropriate number of LVs according to the minimum RMSECV value.

### 3.8. Evaluation Criteria of Models

The model was optimized using samples from calibration sets, and the optimal model was confirmed by the following parameters: Rcv, RMSECV, and RPDCV.

The validation of the predictive ability of the model is based on values of the following indexes: Rc, Rp, RMSEC, RMSEP, RSEC, RSEP, RPDC, and RPDP.

Generally, the optimal model fits the requirement of high R values, small and close RMSE, and RSE values. A model with an RPD value higher than 3 will be considered available for process control [30].

### 3.9. Software

Data acquisition was performed using the OPUS (version 7.0, Bruker Optics Inc., Germany). MATLAB software (version 2018b, Math Works, Natick, MA, USA) was applied to process spectral pretreatment, variable selection, and model construction.

## 4. Conclusions

In this study, four variable selection methods were applied and systematically compared to realize the establishment of a real-time detection system for CQAs of LOS during alcohol precipitation. In order to avoid the influence of abnormal data on data analysis, two abnormal samples were confirmed and excluded by MCCV. SG smoothing was chosen to preprocess the spectra and removed the effect of noise on the dataset. Compared with the commonly applied SIPLS, CARS, and RF variable selection methods, the innovative VCPA-IRIV showed more powerful key variable screening capabilities. Through the precompression of the variable space by VCPA and the accurate calculation of IRIV, the spectral data of geniposide and baicalin were compressed from 2032 to 21 and 13, respectively. The key variables identified by VCPA-IRIV and the variables screened by the other three methods were consistent in the distribution of variable space and were closely related to the structure of substances. Therefore, 1% of the original variables screened by VCPA-IRIV retained the key information of the spectra. By VCPA-IRIV optimization, the RMSECV values of the geniposide and baicalin models decreased from 0.4740 and 0.2951 to 0.1793 and 0.2338, respectively. An external test was introduced to verify the prediction accuracy of the quantitative model. The Rp values of the VCPA-IRIV-PLSR models were higher than 0.95, the RSEP values were lower than 10%, and the RPDP values were higher than two. The model performance parameters proved that the introduction of a variable screening method improved the predictive ability of PLSR for LOS samples while reducing the complexity of the model and enhancing interpretability. The optimal PLSR models can be applied to the quality control of the LOS production process to realize real-time monitoring of CQAs. The system advocated by this study can help researchers to further understand the rules in the LOS production process and provide a reference for the optimization of LOS production quality control.

## Figures and Tables

**Table 1:** Reference values for geniposide and baicalin of the data sets.

Data Sets		Sample Number	Minimum Concentration (mg/mL)	Maximum Concentration (mg/mL)	Mean	STD
Geniposide	Calibration set	74	2.738	7.819	4.965	1.183
	Prediction set	25	3.265	5.675	4.352	0.6560
Baicalin	Calibration set	74	1.763	4.722	3.339	0.7525
	Prediction set	25	2.017	4.610	3.167	0.6825

**Table 2:** Performance of different spectral pretreatments models for 2 indexes.

Pretreatment	LVs ^1^	Rcv	RMSECV ^2^	RPDCV
A. Models for geniposide				
Raw	6	0.9032	0.5045	2.33
Normalization	7	0.9020	0.5074	2.32
SNV	9	0.8962	0.5213	2.25
**SG smoothing**	**10**	**0.9151**	**0.4740**	**2.48**
MSC	9	0.8902	0.5354	2.20
B. Models for baicalin				
Raw	5	0.8847	0.3484	2.15
Normalization	6	0.9121	0.3064	2.44
SNV	7	0.8990	0.3273	2.28
**SG smoothing**	**10**	**0.9187**	**0.2951**	**2.53**
MSC	4	0.8960	0.3319	2.25

^1^ LVs: latent variables; ^2^ RMSECV: root mean square error of cross-validation.

**Table 3:** Calibration results by SIPLS model with different spectral range selection.

NI ^1^	NV ^2^	LVs	Rcv	RMSECV	RPDCV	NV	LVs	Rcv	RMSECV	RPDCV
A. models for geniposide						B. models for baicalin				
10	812	10	0.9630	0.3169	3.71	812	9	0.9208	0.2916	2.56
11	739	10	0.9638	0.3134	3.75	739	8	0.9140	0.3031	2.47
12	676	10	0.9692	0.2896	4.06	677	10	0.9119	0.3067	2.44
13	624	10	0.9667	0.3008	3.91	625	10	0.9264	0.2815	2.66
14	580	10	0.9656	0.3055	3.85	580	10	0.9219	0.2896	2.58
15	541	10	0.9652	0.3074	3.82	540	8	0.9174	0.2974	2.51
16	508	10	0.9762	0.2549	4.61	508	10	0.9212	0.2908	2.57
17	478	10	0.9657	0.3053	3.85	477	10	0.9261	0.2820	2.65
18	451	10	0.9702	0.2846	4.13	451	10	0.9317	0.2714	2.75
19	427	10	0.9673	0.2982	3.94	428	10	0.9299	0.2748	2.72
20	407	10	0.9670	0.2993	3.93	406	10	0.9199	0.2931	2.55
21	388	10	0.9697	0.2869	4.10	386	8	0.9324	0.2701	2.77
22	369	10	0.9687	0.2916	4.03	369	9	0.9293	0.2761	2.71
23	353	10	0.9681	0.2945	3.99	353	9	0.9322	0.2706	2.76
24	338	10	0.9740	0.2664	4.41	339	9	0.9299	0.2748	2.72
25	324	10	0.9715	0.2786	4.22	325	9	0.9390	0.2570	2.91
**26**	**312**	**10**	**0.9783**	**0.2436**	**4.83**	312	9	0.9350	0.2651	2.82
27	300	10	0.9717	0.2775	4.24	300	9	0.9401	0.2548	2.93
28	291	10	0.9755	0.2588	4.54	**289**	**9**	**0.9402**	**0.2546**	**2.93**
29	280	10	0.9700	0.2857	4.11	280	8	0.9380	0.2591	2.88
30	271	10	0.9708	0.2817	4.17	270	10	0.9353	0.2644	2.83

^1^ NI: Number of intervals; ^2^ NV: Number of variables.

**Table 4:** Comparison of results based on different variable selection methods.

Variables Selection Methods	Number of Variables	LVs	Rcv	RMSECV	RPDCV
A. Models for geniposide					
Global	2032	10	0.9151	0.4740	2.48
SIPLS	312	10	0.9783	0.2436	4.83
CARS	76	9	0.9729	0.2716	4.33
RF	100	10	0.9645	0.3103	3.79
**VCPA-IRIV**	**21**	**10**	**0.9883**	**0.1793**	**6.56**
B. Models for baicalin					
Global	2032	10	0.9187	0.2951	2.53
SIPLS	289	9	0.9402	0.2546	2.93
CARS	38	10	0.9382	0.2586	2.89
RF	20	9	0.9452	0.2441	3.06
**VCPA-IRIV**	**13**	**10**	**0.9502**	**0.2329**	**3.21**

**Table 5:** The results of external validation of VCPA-IRIV-PLSR models.

Analytes	LVs	Rc ^1^	RMSEC	RSEC ^2^	RPDC ^3^	Rp ^4^	RMSEP	RSEP ^5^	RPDP ^6^
Geniposide	10	0.9939	0.1297	2.54%	9.06	0.9654	0.1676	3.81%	3.84
Baicalin	10	0.9629	0.2018	5.90%	3.70	0.9597	0.1880	5.81%	3.57

^1^ Rc: Correlation coefficients of calibration; ^2^ RSEC: Relative standard error of calibration; ^3^ RPDC: Residual predictive deviation of calibration; ^4^ Rp: Correlation coefficients of prediction; ^5^ RSEP: Relative standard error of prediction; ^6^ RPDP: Residual predictive deviation of prediction.

## Data Availability

Not applicable.

## Supplementary Materials

The following supporting information can be downloaded at: https://www.mdpi.com/article/10.3390/molecules28010004/s1, Figure S1: HPLC chromatograms of standard solution and LOS sample (1. geniposide, 2. baicalin).

## Sample Availability

Samples of the compounds are available from the authors.
