# Supplementary figures and images for: Rapid Determination of Geniposide and Baicalin in Lanqin Oral Solution by Near-Infrared Spectroscopy with Chemometric Algorithms during Alcohol Precipitation
Source: Molecules. 2022 Dec 20;28(1):4. doi: 10.3390/molecules28010004 (PMC9822193; doi:10.3390/molecules28010004)

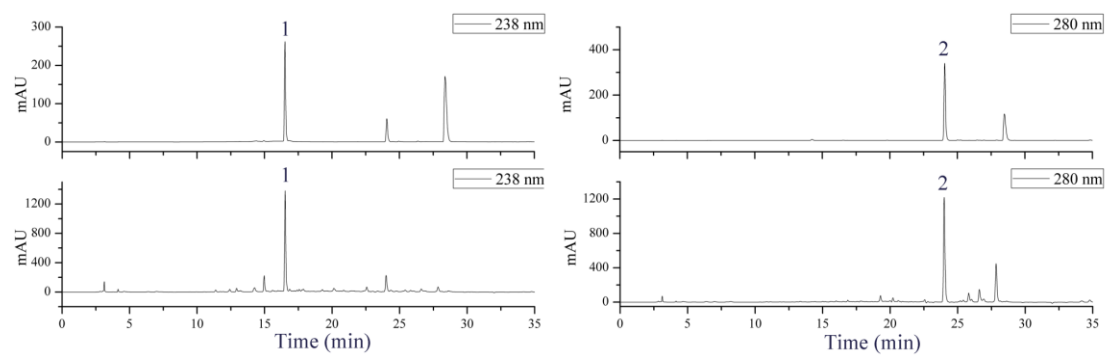

**Figure S1** HPLC chromatograms of standard solution and LOS sample (1. geniposide, 2. baicalin).

Supplement: Supplementary file 1 [file molecules-28-00004-s001.zip › molecules-2066950-supplementary.pdf]
